# Clinical Profile and Outcome of Conservatively Managed Emphysematous Pyelonephritis

**DOI:** 10.5402/2012/931982

**Published:** 2012-03-18

**Authors:** Praveen Kumar Kolla, Desai Madhav, Satish Reddy, Suneetha Pentyala, Panil Kumar, Rama Mohan Pathapati

**Affiliations:** ^1^Department of Nephrology, Narayana Medical College Hospital, Chinthareddypalem, Andhra Pradesh, Nellore 524002, India; ^2^Department of Radiology, Narayana Medical College Hospital, Chinthareddypalem, Andhra Pradesh, Nellore 524002, India; ^3^Department of Clinical Pharmacology, Narayana Medical College Hospital, Chinthareddypalem, Andhra Pradesh, Nellore 524002, India

## Abstract

Emphysematous pyelonephritis (EPN) is a severe, necrotizing renal parenchymal infection characterized by production of intraparenchymal gas. EPN predominantly affects female diabetics and immunocompromised patients. In a three-year period 2008–2011, a total of 8 patients were admitted to our hospital. All of them were diabetics, and both males and females were equally affected. These patients showed vague symptoms at admission and frequently presented with fever, loin pain, dysuria, and pyuria necessitating urgent medical attention. EPN required radiological diagnosis. CT scan revealed bilateral EPN with urinary obstruction and hydronephrosis in 50% of patients. *Escherichia coli* was found to be the causative organism in all the patients. Treatment comprised of resuscitation, normalization of serum electrolytes and blood sugars, administration of parenteral antibiotics, and relieving ureteric obstruction if present. All the patients improved with conservative management without any mortality.

## 1. Introduction

Emphysematous pyelonephritis (EPN) is a life-threatening, fulminant, necrotizing upper urinary tract infection associated with gas within the kidney and/or perinephric space [1]. We present the clinical profiles and outcome of eight patients with EPN who were managed conservatively without any mortality.

## 2. Methods

During the period 2008–2011, a total of eight diabetic patients with clinical features of EPN and computerized tomography (CT) image showing gas in renal parenchyma, collecting system or perirenal space, and no fistulous connection between urinary tract and bowel were evaluated.

## 3. Results

**Figure 1:**
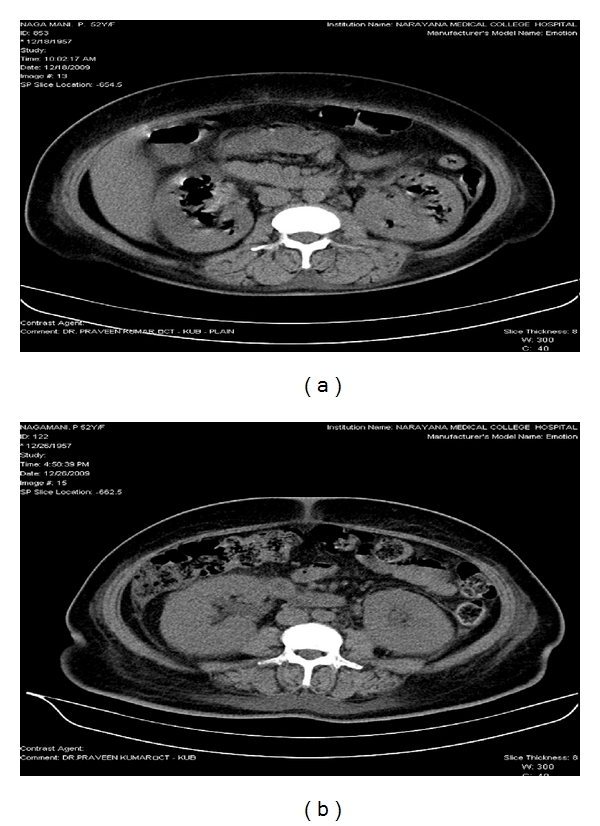
(a) CT KUB showing bilateral emphysematous pyelonephritis (Class IV). (b) CT KUB showing resolution of air pockets after 10 days of treatment.

**Figure 2:**
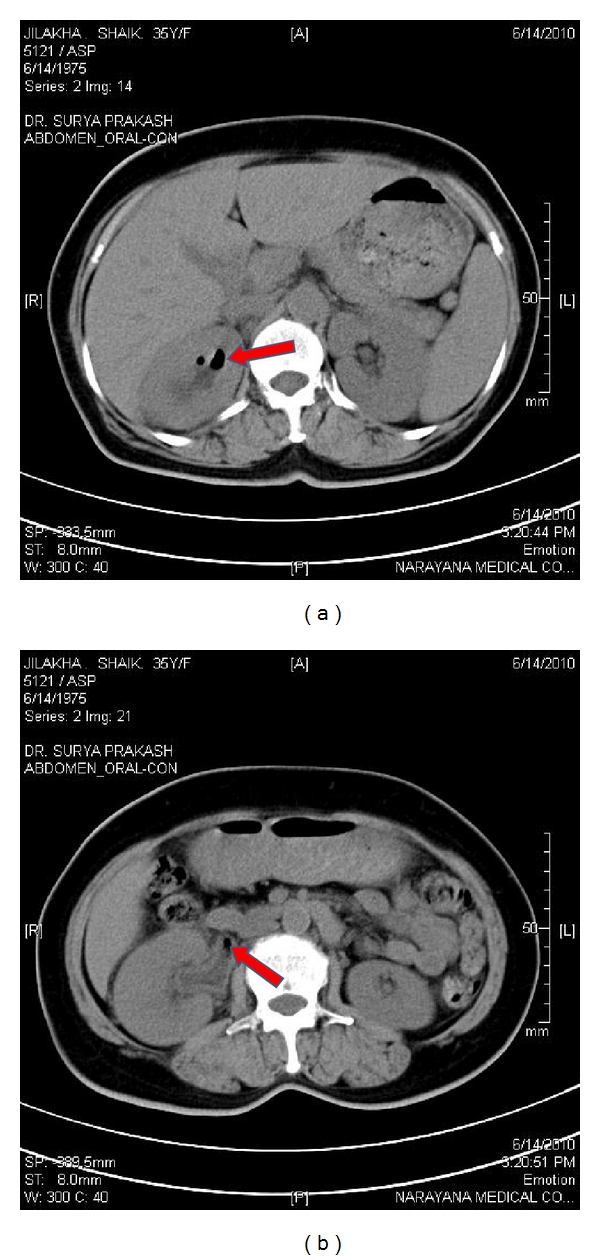
(a) CT KUB showing air pockets in right collecting system. (b) CT KUB showing air pocket in right proximal ureter.

**Figure 3:**
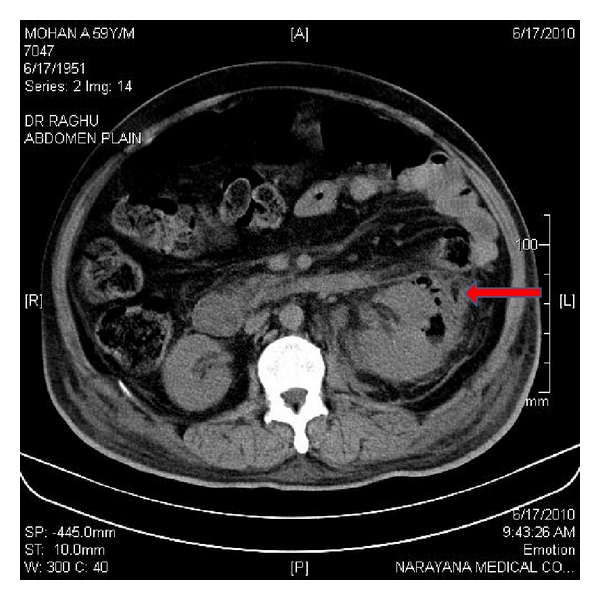
CT KUB showing air pocket in left renal parenchyma and perinephric collection.

**Figure 4:**
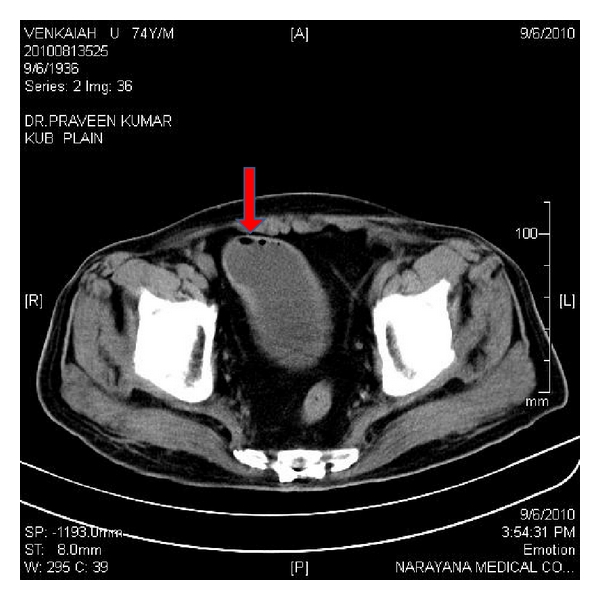
CT KUB showing emphysematous cystitis.

All the patients were between 33 and 58 yrs. Males and females were equal in numbers. Clinical profiles and outcomes were shown in Table 1. All of them presented with fever, loin pain, dysuria, and oliguria. At admission, renal dysfunction was observed in all these patients with a mean serum creatinine of 5.47 ± 2.3 (2.3–7.8) mg/dL. Most of them had poorly controlled blood sugar levels (seven out of eight). *Escherichia coli* was found to be the causative organism in all the patients as per urine culture. CT imaging showed bilateral involvement in four (Huang et al. staging class IV) and unilateral in 4 (Classes I, II and III). Hydronephrosis was seen in fifty percent of patients (three unilateral and one bilateral). There were air pockets in the perinephric collection, proximal ureter, and in the urinary bladder of two patients (Figures 1, 2, 3, and 4). Five patients required renal replacement therapy for severe renal failure. DJ stenting was done in 4 patients for obstructed urinary system. None of them required any other surgical intervention. All patients were discharged in a stable condition with a mean serum creatinine of 2.40 ± 0.76 (1.6–3.3) mg/dL.

## 4. Discussion

EPN is defined as a severe necrotizing renal parenchymal infection that is characterized by the bacterial production of gas within the renal parenchyma. The commonest causative organism is *Escherichia coli*, followed by *Proteus*, *Klebsiella*, anaerobic *Streptococci*, and *Candida*. EPN is common in females, diabetics [1], and immune-compromised patients [2]. EPN has been reported in renal transplant recipients [3–6] and in patients with polycystic kidney disease [7, 8]. The diagnosis of EPN is often delayed because the appearances of symptoms are very hazy and nonspecific. Common presentation includes fever, abdominal pain, dysuria, vomiting, depressed level of consciousness, shock, renal angle tenderness, and acute kidney injury [9]. Less common presentation includes dyspnea, crepitus over the flanks, and pneumaturia [10].

In our study, all the patients were diabetics, and majority had uncontrolled blood sugars. All of them presented with fever, loin pain, dysuria, pyuria, and oliguria. Dyspnea and pedal edema are seen in 5/8, and one had altered sensorium. The causative organism was found to be *Escherichia coli* in urine cultures in all the patients. None of the patients had mixed infections.

EPN requires a radiological diagnosis and CT being the definitive modality for diagnosis [11]. CT imaging [12] not only reveals the presence and position of gas in the renal parenchyma but also is useful to assess the extent of renal parenchymal involvement and response to therapy. Abdominal ultrasonography (USG) and X-ray can also be used to diagnose EPN. In our study, ultrasonography gave a clue for the presence of gas in renal parenchyma in five patients. CT showed the presence of EPN, even in those situations where USG could not pick up. Urinary obstruction and hydronephrosis was noted in 50% of patients. It is possible that urinary tract obstruction and decreased renal vascular supply due to diabetes might have contributed for the development of EPN in our patients.

Patients with emphysematous pyelonephritis (EPN) should be treated with aggressive medical management and possibly prompt surgical intervention [1, 13, 14]. Active management with appropriate parenteral antibiotics, addressing the fluid-electrolyte and hemodynamic status, stringent control of diabetes with insulin, and relieving ureteric obstruction either by percutaneous drainage or internal stenting has improved the clinical outcome of our patients without any mortality. None of them required any major surgical intervention, even though more than half of the patients were in class 3 and class 4 EPN and having more than two risk factors such as elevated serum creatinine, altered sensorium, and shock. Recent case reports have also described successful outcome in patients with bilateral EPN with medical therapy alone [15, 16]. At discharge, all the patients had elevated serum creatinine; time to normalization of renal parameters could not be assessed as most of these patients were lost for followup.

## 5. Conclusions

Emphysematous pyelonephritis is not uncommon. It should be suspected in every diabetic patient with urosepsis. CT is the definitive modality for diagnosing EPN. Early diagnosis and effective conservative treatment obviate the need for nephrectomy and decrease mortality.

## Figures and Tables

**Table 1:** Clinical profile and outcome of patients with EPN.

Patient	1	2	3	4	5	6	7	8
Gender/age (yr)	M/54	F/52	M/33	F/53	F/55	M/58	M/38	F/54
Duration of diabetes (yr)	18	10	4	5	8	12	5	6
Fever	+	+	+	+	+	+	+	+
Loin pain	+	+	+	+	+	+	+	+
Oliguria	+	+	−	+	−	+	+	+
Dysuria	+	+	+	+	+	+	+	+
Serum creatinine at admission (mg/dL)	7	7.7	2.3	7.8	2.7	5.8	3.1	7.4
Serum creatinine at discharge (mg/dL)	3.3	3.24	1.8	2.6	1.6	1.7	1.8	3.2
Urine culture	*E. coli *	*E. coli*	*E. coli*	*E. coli*	*E. coli*	*E. coli *	*E. coli *	*E. coli *
Blood culture	Sterile	Sterile	Sterile	Sterile	Sterile	Sterile	Sterile	Sterile
Laterality	Bilateral	Bilateral	Unilateral	Bilateral	Unilateral	Bilateral	Unilateral	Unilateral
Huang et al. CT-scan staging	4	4	3A	4	1	4	2	2
Hemodialysis sessions	4	4	0	3	0	4	0	4
Hydronephrosis	Yes	No	No	Yes	No	Yes	No	Yes
DJ stent	Yes	No	No	Yes	No	Yes	No	Yes
Duration of DJ stent (days)	10	−	−	14	−	14	−	12
Recurrence of obstruction	No	−	−	No	−	No	−	No
Antibiotics used	IME	IME	IME	Pip + Tz	Cef + Slb	IME	Cef + Slb	IME
Duration of antibiotics (days)	10	14	10	14	14	10	14	10

IME: Imipenem, Pip + Tz: Piperacillin + Tazobactam, Cef + Slb: Cefoperazone + Sulbactam.
